# Mesenchymal-Stem-Cell–Derived Extracellular Vesicles Mitigate Trained Immunity in the Brain

**DOI:** 10.3389/fbioe.2020.599058

**Published:** 2020-11-19

**Authors:** Yiwei Feng, Min Guo, Hongchen Zhao, Sida Han, Qiang Dong, Mei Cui

**Affiliations:** ^1^Department of Neurology, Huashan Hospital, Fudan University, Shanghai, China; ^2^State Key Laboratory of Medical Neurobiology, MOE Frontiers Center for Brain Science, Department of Neurology, Huashan Hospital, Fudan University, Shanghai, China

**Keywords:** trained immunity, stroke, Alzheimer’s disease, MSC (1991) 49N10, MSC-derived EVs

## Abstract

Trained immunity was recently discovered in innate immune cells and shown to facilitate the clearance of pathogens at the time of occurrence of the second insult. However, it exacerbates several aspects of neuropathologies, and proper therapy is needed to rectify this abnormal immune reaction. Mesenchymal-stem cells (MSCs) exhibit a distinct capability for brain repair but are associated with safety concerns. Extracellular vesicles derived from MSCs are a promising alternative therapy. In this study, we used lipopolysaccharides to activate trained immunity in the brain and examined the therapeutic potential of MSC-derived extracellular vesicles in mitigating the trained-immunity-induced exacerbated neuropathology. We found that MSC-derived extracellular vesicles showed comparable effects to those of MSCs in the mitigation of trained immunity in the brain. Moreover, the administration of MCS-derived extracellular vesicles mitigated the aggregated inflammatory responses in the acute stage of stroke and alleviated the trained-immunity-induced increased load of amyloid-β in APP/PS1 mice. We further investigated the molecular machinery of MSC-derived extracellular vesicles and found that IL-10 is important for the mediation of the therapeutic potential of MSC-derived extracellular vesicles toward the alleviation of trained immunity. Our study indicates that extracellular-vesicle-based regenerative strategies might be useful to mitigate trained immunity in the brain.

## Introduction

Immune memory has long been believed to exist only in the adaptive immune system. However, recent evidence indicates that innate immune cells also display memory effects, namely trained immunity (Netea et al., 2015, 2016; Wendeln et al., 2018). For example, certain inflammatory stimuli, such as lipopolysaccharides (LPS), prime peripheral monocytes, leading them to respond more efficiently when the second inflammatory insult occurs (Biswas and Lopez-Collazo, 2009; Saeed et al., 2014). Trained immunity triggers the more efficient clearance of pathogens by myeloid cells. However, in the central nervous system (CNS), microglia, which are the brain-resident macrophages, exhibit a different aspect of trained immunity. It has been demonstrated that mice subjected to initial inflammatory stimuli, such as LPS, exhibit activation of the innate immune memory in the brain, which exacerbated stroke prognosis and deteriorated the pathology of Alzheimer’s disease in the long term (Wendeln et al., 2018). The loss of dopaminergic neurons and aggregated α-synuclein are also prominent in an MPTP-induced model of Parkinson’s disease (PD) after a single LPS injection (Qin et al., 2007). Once activated, trained immunity appears as a long-lasting effect that modulates the progression of many brain diseases. Therefore, adequate therapies are urgently needed to rectify this abnormal immune status in the CNS.

Advances in regenerative medicine have revealed the great potential of mesenchymal-stem cells (MSCs) in brain repair based on their remarkable anti-inflammatory and immunomodulatory properties (Galindo et al., 2011; Sherman et al., 2019). Our previous study found that MSCs triggered the remission of trained immunity through the inhibition of H3K4 methylation (Feng et al., 2020). However, concerns regarding their safety as well as their tumor- or microinfarction-inducing potential limit the translational capacity of MSCs (Kunter et al., 2007).

Mesenchymal-stem cells are potent producers of extracellular vesicles (EVs). These vesicles (50–1000 nm in size) contain substantial components that can promote tissue repair and mediate MSC paracrine function (El Andaloussi et al., 2013; Ha et al., 2016). As MSCs cannot cross the blood−brain barrier (BBB) and most MSCs injected show a lung-predominant distribution, MSC-derived EVs may represent an alternative option as they could reach the brain parenchyma and, to a large extent, mimic the regenerative and immunosuppressive effects of MSCs (Wood et al., 2011; Wang et al., 2019).

The vast research efforts dedicated to the elucidation of the anti-inflammatory mechanism of MSCs identified IL-10 as an important mediator of the switch of the macrophage phenotype from the M1 (proinflammatory) to the M2 (anti-inflammatory) types and as a trigger of the MSC-induced reparative process (Eirin et al., 2017; Park et al., 2019). However, whether the protective effect of MSCs or MSC-derived EVs via the remission of trained immunity is dependent on an IL-10-mediated mechanism remains elusive.

In this study, we tested the therapeutic effect of MSC-derived EVs on trained immunity in the brain and further examined this process in the context of two neurological diseases in which the relative pathology is substantially exacerbated by trained immunity. Moreover, we studied whether this protective effect was dependent on an IL-10-mediated mechanism.

## Results

### Characteristics of MSC-Derived EVs

**FIGURE 1:**
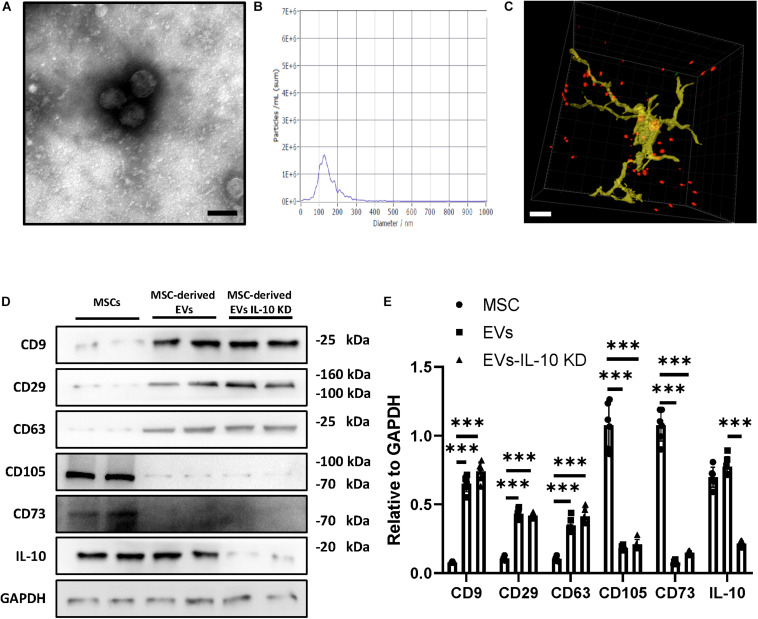
Characteristic of MSC-derived EVs. **(A)** Representative TEM images of MSC-derived EVs. Scale bar, 100 nm. **(B)** NTA analysis of MSC-derived EVs. **(C)** Representative 3-dimensional confocal images showing microglia (Iba1, green) engulfing MSC-derived EVs (red). Scale bar, 10 μm. **(D)** Immunoblot **(D)** and quantitative analysis **(E)** of CD9, CD29, CD63, CD105, CD73, IL-10, GAPDH in MSCs, MSC-derived EVs, and MSC-derived EVs with IL-10 knockdown. Data are presented as the mean ± standard deviation (***p < 0.001).

Ultracentrifugation was used to isolate MSC-derived EVs in our study. The isolated exosomes were observed to exhibit spherical structures via transmission electron microscopy (Figure 1A), and were mainly distributed among diameters of 100−150 nm, as assessed by nanoparticle tracking analysis (Figure 1B). Moreover, we validated the expression of key proteins in our isolated EVs by identifying the expression of characteristic EV and MSC markers, such as CD9, CD29, and CD63 (Figures 1D,E). In addition, IL-10 knockdown significantly inhibited IL-10 expression in MSC-derived EVs, as shown in Figure 1D. Next, we labeled EVs with a red fluorescent dye and injected the labeled EVs into the tail vein of mice. Thirty minutes after the injection, mice were sacrificed and EVs were tracked in frozen sections of the brain. We detected the distribution of EVs across the brain parenchyma and the internalization of MSC-derived EVs by microglia (Figure 1C). These results suggest that peripherally administered MSC-derived EVs were able to cross the BBB and were engulfed by microglia in the CNS.

### MSC-Derived EVs Attenuate the Innate Immune Memory in the Brain

It has been established that inflammation in the periphery can prompt immune responses in the brain, and that peripheral low-dose lipopolysaccharide (LPS) stimulation can induce microglial immune memory (Wendeln et al., 2018). As MSC-derived EVs were able to cross the brain parenchyma and affect microglia, we wondered whether MSC-derived EVs could alleviate the innate immune memory and whether this alleviation was dependent on an IL-10 mediated mechanism.

**FIGURE 2:**
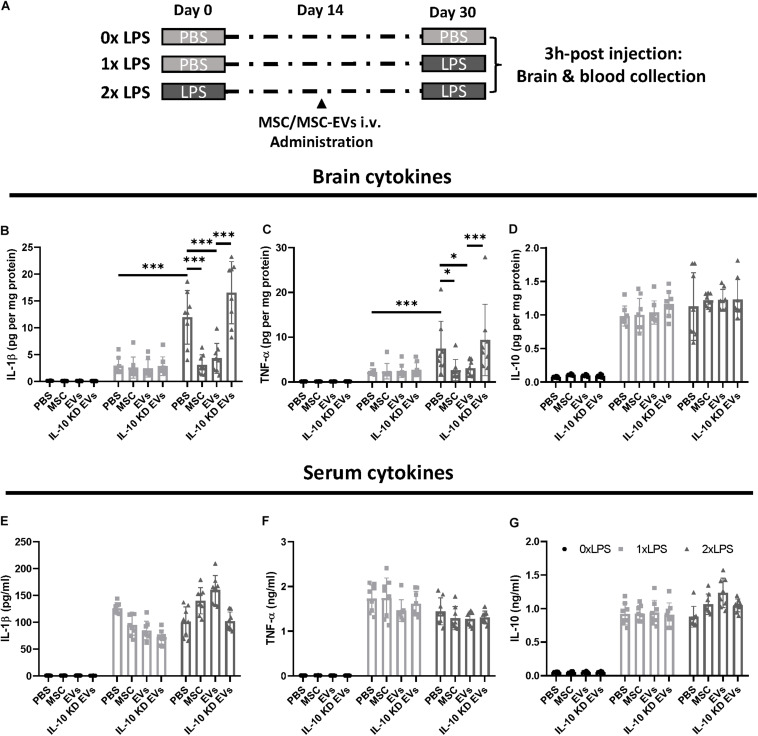
MSC-derived EVs mitigated LPS-induced trained immunity. **(A)** Schematic diagram of the experimental design. **(B–D)** Quantitative ELISA analysis of cytokine profiles in brain parenchyma following LPS administration (*n* = 8 mice per group). **(E–G)** Quantitative ELISA analysis of cytokine profiles in peripheral blood following LPS administration (*n* = 8 mice per group) Data are presented as the mean ± standard deviation (^∗^*p* < 0.05; ^∗∗∗^*p* < 0.001).

First, we established a mouse model in which the innate immune memory was activated by low-dose LPS administration. And these mice were re-stimulated using PBS or LPS (1× or 2× LPS) 30 days after the initial LPS stimulation, to examine the inflammatory responses in the brain or peripheral blood serum (Figure 2A). We found that the secondary LPS stimulation (both 1× and 2× LPS) exacerbated brain cytokine production, as evidenced by the significantly increased levels of pro-inflammatory and anti-inflammatory cytokine IL-1β, TNF-α, and IL-10 (Figures 2B,C) and 2× LPS mice showed a significant elevation of pro-inflammatory cytokines IL-1β, TNF-α compared with 1× LPS mice. These results indicate the successful establishment of innate immune memory in the brain.

We further tested the therapeutic potential of MSC-derived EVs regarding the remittance of these arrogated inflammatory responses. MSC-derived EVs (2 × 10^9^ NVs) were intravenously injected at day 14 after the first LPS stimulation. MSCs (1 × 10^7^/ml, 0.5 ml per mouse) were injected at the same time point in a separate group, to compare the therapeutic effect of MSC-derived EVs with that of MSCs regarding immune training. At day 30, the exacerbated inflammatory responses that were observed in mice re-stimulated with LPS (2× LPS) were diminished in mice subjected to MSC-derived EV and MSC administration; however, the alleviation afforded by MSC-derived EVs was abolished by IL-10 knockdown in these EVs (Figures 2B–D). Interestingly, neither MSCs nor MSC-derived EVs affected the levels of inflammatory cytokines in the serum of mice stimulated with both 1× and 2× LPS (Figures 2E–G).

Together, these results indicate that MSC-derived EVs can penetrate into the CNS and alleviate the innate immune memory in the brain to an extent comparable to MSC administration.

### MSC-Derived EVs Rescued the Deteriorated Neuropathology Caused by Immune Training

The results reported above indicated the therapeutic potential of MSC-derived EVs in remitting the innate immune memory in the brain. Next, we examined this therapeutic effect of MSC-derived EVs on various disease pathologies in the CNS that are greatly affected by the innate immune memory.

**FIGURE 3:**
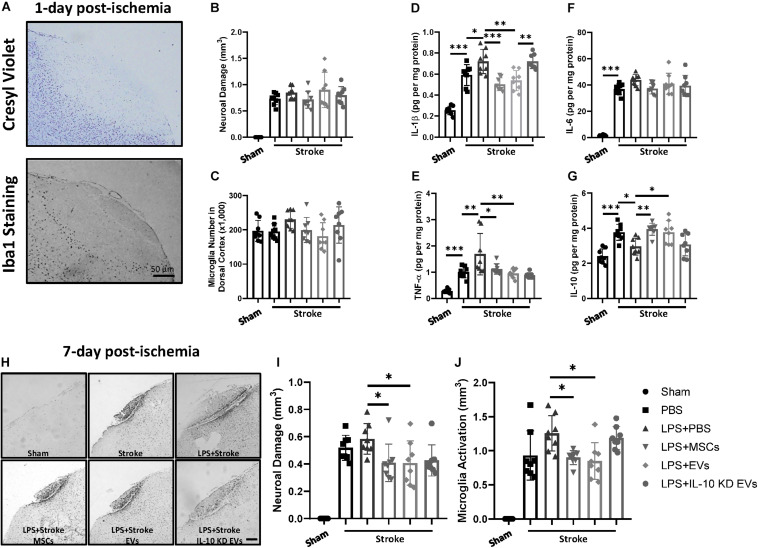
The exacerbated stroke pathology caused by trained immunity was mitigated by MSC-derive EVs. **(A)** Representative cresyl violet staining and Iba1 IHC staining of the ischemic brain cortex at 1 day post-ischemia. Scale bar: 50 μm. **(B,C)** Quantitative analysis of neuronal damage **(B)** (cresyl violet, *n* = 8 mice per group) and number of microglia in the dorsal cortex **(C)** (Iba1 IHC staining, *n* = 8 mice per group) at 1 day post-ischemia. **(D–G)** Quantitative ELISA analysis of cytokine profiles in the ischemic cortex at 1 day post-ischemia (*n* = 8 mice per group). **(H)** Representative IHC staining of Iba1-positive microglia in the ischemic cortex at 7 days post-ischemia. Scale bar: 50 μm. **(I,J)** Quantitative analysis of infarct volume **(I)** and microglial activation **(J)** at 7 days post-ischemia (*n* = 8 mice per group). Data are presented as the mean ± standard deviation (*p < 0.05; **p < 0.01; ***p < 0.001).

It has been previously demonstrated that low-dose LPS stimulation exacerbated stroke prognosis 1 month after exposure to this agent, and that this effect was dependent on the innate immune memory (Wendeln et al., 2018). Wild-type mice were injected with single LPS, and focal ischemia was induced 1 month later. At 1-day post-ischemia, neuronal damage and microglial density were undistinguishable among the various treatment groups (Figures 3A–C). However, an aggregated inflammatory response was observed in mice that were pre-injected with LPS, showing as elevated IL-1β, IL-6, and TNF-α and decreased IL-10 (Figures 3D–G). Both MSCs and MSC-derived EVs abolished this increased production of proinflammatory cytokines and rescued the production of IL-10. However, knockdown of IL-10 in MSC-derived EVs abolished this alleviation (Figures 3D–G). At 7-day post-ischemia, we observed that although LPS treatment did not increase the infarct size, MSCs and MSC-derived EVs significantly alleviated the infarct volume when compared with LPS + PBS group; however, knockdown of IL-10 in MSC-derived EVs failed to abolish this therapeutic effect (Figures 3H–J).

**FIGURE 4:**
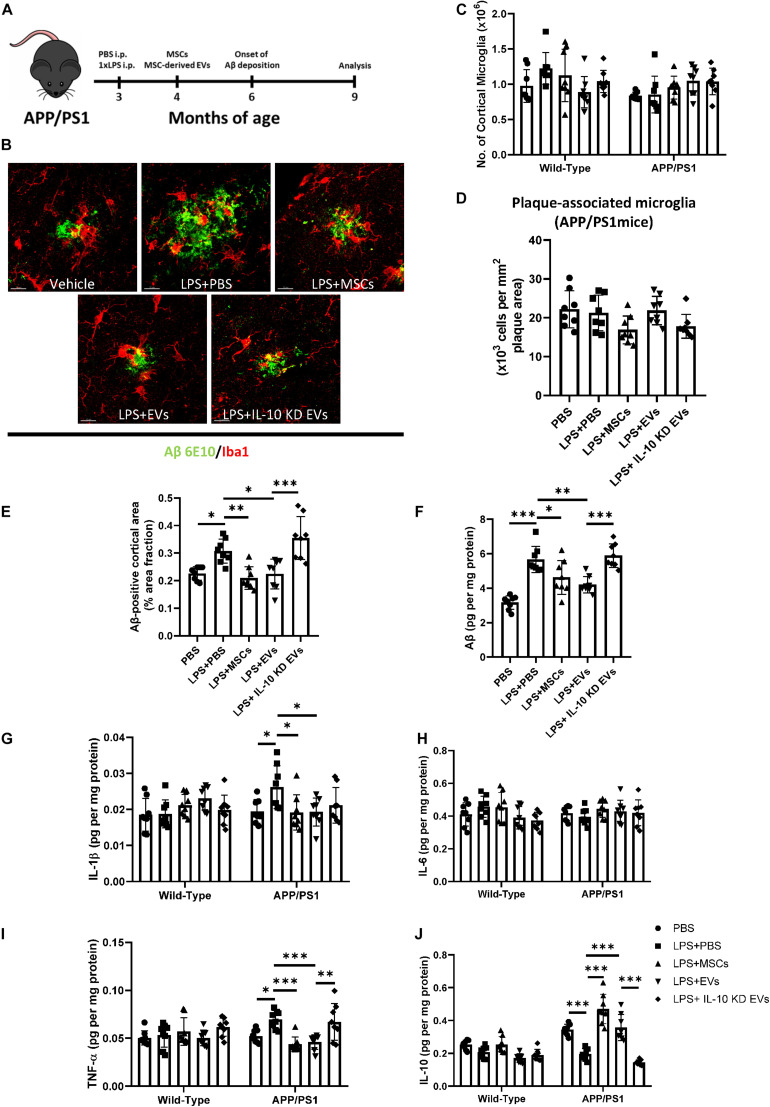
The exacerbated β-amyloidosis caused by trained immunity was mitigated by MCS-derived EVs. **(A)** Schematic diagram of the experimental design. **(B)** Representative immunofluorescence images showing Aβ deposition (green) and surrounding Iba1 (red). Scale bar: 5 μm. **(C,D)** Quantitative analysis of total cortical **(C)** and plaque-associated **(D)** microglia (*n* = 8 mice per group). **(E,F)** Quantitative analysis of cortical Aβ plaque load **(E)** (*n* = 8 mice per group) and Aβ protein levels by ELISA **(F)** (*n* = 8 mice per group). **(G–J)** Quantitative ELISA analysis of cytokine profiles in wild-type and APP/PS1 mice at 9 months (*n* = 8 mice per group). Data are presented as the mean ± standard deviation. (*p < 0.05; **p < 0.01; ***p < 0.001).

Next, we tested the therapeutic potential of MSC-derived EVs in a second disease model. It has been established that the innate immune memory promotes the pathological progression of Alzheimer’s disease (AD) (Wendeln et al., 2018). The model consists in a single injection of low-dose LPS (1× LPS) into 3-month-old APP23 mice, which exacerbates the formation of plaques of insoluble amyloid-β (Aβ) at the age of 9 months (Figure 4A). Based on this model, trained immunity was induced by low-dose LPS administration to 3-month-old APP23 mice, and MSC/MSC-derived EVs were injected intravenously 1 month later. A pathological analysis was performed in 9-month-old APP23 mice (Figure 4B). Although neither 1× LPS administration nor MSCs/MSC-derived EVs/MSC-derived EVs with IL-10 knockdown affected the number of cortical microglia or plaque-associated microglia (Figures 4C,D), 1× LPS significantly increased the plaque load and total Aβ levels compared with control animals (Figures 4E,F). MSCs and MSC-derived EVs yielded a similar alleviation of the plaque load and total Aβ levels, whereas MSC-derived EVs with IL-10 knockdown abolished the therapeutic effect of MSC-derived EVs on AD pathology (Figures 4E,F). Moreover, MSCs and MSC-derived EVs mitigated the upregulation of the proinflammatory TNF-α and IL-1β in 1× LPS-stimulated 9-month-old APP23 mice (Figures 4G–J). Finally, MSCs and MSC-derived EVs rescued the inhibited IL-10 production in 1× LPS-stimulated 9-month-old APP23 mice and MSC-derived EVs with IL-10 knockdown abolished this therapeutic effect (Figures 4G–J). Therefore, it could be observed that the exacerbated AD pathology caused by trained immunity was mitigated by MSC-derived EVs via an IL-10 dependent mechanism.

## Discussion

In this study, we uncovered the therapeutic potential of MSC-derived EVs in mitigating trained immunity in the CNS. The initial LPS training exacerbated the inflammatory responses caused by the secondary LPS challenge administered 1 month later, and MSC-derived EVs remitted this aggregated response via an IL-10-dependent mechanism. We further examined this therapeutic potential of MSC-derived EVs in two neurological models in which the pathological changes were caused by trained immunity. We found that the exacerbated inflammatory responses resulting from LPS training in the acute phase of stroke were remitted by MSCs or MSC-derived EVs. In addition, MSCs or MSC-derived EVs decreased the infarct volume at 7 days after ischemic stroke. Moreover, the exacerbated deposition of amyloid-β plaques triggered by LPS training was also remitted by MSCs or MSC-derived EVs.

The participation of trained immunity in the development of neurological pathologies has been discovered recently (Wendeln et al., 2018). Once activated by a proinflammatory stimulus, such as LPS, oxLDL, DAMPs, or TNF-α, microglia undergo an epigenetic reprogramming that increases the H3K4 methylation or H3K27 acetylation levels in inflammation-related genes, resulting in a stronger response to secondary stimuli upon re-challenge (Crişan et al., 2016). In contrast with the function of trained immunity in the periphery, where it clears pathogens more efficiently, trained immunity has detrimental effects in the CNS (Netea et al., 2016). This may be attributed to the longevity of microglia and the vicious cycle in which the presence of insoluble aggregated proteins, such as Aβ and α-synuclein, activates microglia, with trained microglia further exacerbating these pathologies through enhanced inflammatory responses (Qin et al., 2007; Wendeln et al., 2018). As a result, the activation of trained immunity deteriorates stroke prognosis, exacerbates the pathologies of AD, and promotes the aggregation of α-synuclein in PD in the long term (Sacino et al., 2013; Rutherford et al., 2015; Wendeln et al., 2018).

Regarding this long-term effect of trained immunity, we should pay attention to the factors that can initiate it in the brain. It could be noted that trained immunity can be activated not only by LPS, but also by many high-risk factors that deteriorate the microenvironment of the brain parenchyma. For example, the western diet can induce epigenetic reprogramming of the innate immune system in a NLRP3-dependent manner (Christ et al., 2018). Moreover, hypertension exacerbates the pathologies of amyloid-β and worsens stroke prognosis, which is related to the priming of microglia (Perry and Holmes, 2014; Shen et al., 2015). It is expected that other risk factors, such as atherosclerosis and diabetes, also exacerbate neuropathologies through the activation of trained immunity (Perry and Holmes, 2014). Finally, proper therapeutics aim to rectify this fatal effect of trained immunity in the CNS.

Our previous study found that MSCs could alleviate trained immunity through the inhibition of H3K4 methylation (Feng et al., 2020). Although MSC therapy shows potential regarding the mitigation of neuropathologies, the main distribution of MSCs after their intravenous injection is mostly restricted to the lungs, indicating that the therapeutic effect of MSCs occurs mainly via a paracrine-mediated mechanism (Gnecchi et al., 2016; Sherman et al., 2019). Therefore, MSC-derived EVs can be an alternative method to execute the therapeutic potential of MSCs, as MSC-derived EVs share a comparable therapeutic component with the parental MSCs and can easily cross the blood brain barrier (Zagrean et al., 2018). Moreover, MSC-derived EVs, as a cell-free therapy, can overcome the limitations of MSC therapy, such as safety and tumor induction.

The therapeutic effects of MSC-derived EVs on many pathological processes have been demonstrated and are mediated by vesicular membrane proteins, cytosolic proteins, mRNAs, and miRNAs (Phinney and Pittenger, 2017; Yin et al., 2019). In particular, EV-associated proteins are critical in the mediation of the therapeutic potential of MSC-derived EVs. The anti-inflammatory component of MSC-derived EVs has been shown to promote brain repair in mice with ischemic stroke and other neurological diseases (Yang et al., 2017; Chen and Chopp, 2018; Hong et al., 2018; Mendt et al., 2019). Here, we found that MSC-derived EVs alleviated trained immunity and remitted the associated exacerbation of neuropathologies. In a model of focal ischemia, the proinflammatory cytokines that were released in the acute stage of stroke were upregulated by LPS training, and this effect was abolished by MSC-derived EVs. We also evaluated the therapeutic potential of MSC-derived EVs in a model of AD. Notably, LPS training significantly increased the plaque load of insoluble amyloid-β, whereas MSC-derived EVs significantly remitted this exacerbation.

In addition to the direct therapeutic effect of MSC-derived EVs, we investigated the plausible mediator of their effects. The production of the anti-inflammatory cytokine IL-10 is one of the most important mechanisms evolved by many immune cells to counteract the damage driven by excessive inflammation (Strle et al., 2001; Couper et al., 2008; Lobo-Silva et al., 2016). Binding of IL-10 to its receptor activates a series of signaling cascades that are mediated by the JAK and STAT pathway and regulate several steps of the immune response, such as decreasing the expression of genes encoding inflammatory cytokines and preventing apoptosis (Riley et al., 1999). IL-10 in MSC-derived EVs has been shown to polarize macrophages from the proinflammatory M1 to the anti-inflammatory M2 type and to attenuate kidney inflammation and bacteria-induced sepsis (Eirin et al., 2017). Here, we found that MSC-derived EVs expressed IL-10 at a level that was comparable to that of their parent MSCs, and that the administration of MSC-derived EVs also upregulated IL-10 in the infarct region and brain parenchyma in APP/PS1 mice. Upon knockdown of IL-10 in MSC-derived EVs, they failed to exert their protective effect of remitting trained immunity and mitigating the associated exacerbation of the neuropathology caused by trained immunity, thus underscoring the role of this anti-inflammatory cytokine in the mediation of the alleviation of trained immunity by EV administration.

Although we found that MSC-derived EVs mitigated trained immunity, further research is needed to clarify the molecular machinery underlying this phenomenon. Moreover, the current knowledge about trained immunity in the CNS focuses mainly on its mechanism and its relationship with brain diseases. There is an urgent need to find a proper strategy to detect this malfunction in the CNS. We believe that the development of such a detection method will allow MSC-derived EVs to rectify this abnormal activation of trained immunity in the CNS, thus promoting the development of an EV-based cell-free therapy.

## Materials and Methods

### Animals

C57BL/6J male mice (3 months of age) were purchased from Charles River, and APPswe/PS1dE9 (APP/PS1) mice (3 months of age) were purchased from Guangdong Medical Experimental Animal Center. Animals were housed in the Experimental Animal Center of Fudan University in a temperature- and humidity-controlled specific-pathogen-free laboratory with a 12 h/12 h light/dark cycle. All procedures were performed in accordance with the Guide of the National Science Council of the People’s Republic of China, and the study was approved by the Ethics Committee of Fudan University, Shanghai, China (IRB approval number 20190972A259). This manuscript was written in accordance with the Animal Research: Reporting of *In Vivo* Experiments (ARRIVE) guidelines.

### Peripheral Immune Stimulation

Three-month-old mice were randomly assigned to treatment groups and were injected intraperitoneally (i.p.) with bacterial lipopolysaccharides (LPS from *Salmonella enterica* serotype typhimurium; Sigma) at a dose of 500 μg/kg of bodyweight or with vehicle (PBS). In the 2× LPS group, mice were re-stimulated with the same dose of LPS 1 month after the initial injection.

At the specified time points, animals were deeply anesthetized using sedaxylan and ketamine (64 and 472 mg/kg, respectively), blood was collected from the right ventricle of the heart, and animals were transcardially perfused with ice-cold PBS through the left ventricle. The brain was removed and sagitally separated into the two hemispheres, which were either fixed in 4% paraformaldehyde (PFA) or fresh frozen on dry ice. Fresh-frozen hemispheres were homogenized using a Cell Lysis Buffer (Invitrogen) containing phosphatase and protease inhibitors (Thermo Fisher Scientific). Fixed hemispheres were kept in 4% PFA for 24 h, dehydrated in 30% sucrose in PBS, frozen in 2-methylbutane, and coronally sectioned at 25 μm using a freezing-sliding microtome (Leica).

### Isolation of MSCs and MSC-Derived EVs

Umbilical cords were sampled from healthy women who underwent delivery via cesarean section. Each donor had been confirmed not to have infectious diseases, pregnancy complications, HBV, HIV, or syphilis. Approval was granted by the donors, and all procedures were in accordance with the guideline of the Medical Ethics Committee of the Health Bureau.

The isolation of hUCMSCs was performed as described previously. Briefly, the umbilical cord was placed in ice-cold PBS, and the arteries and veins of the umbilical cord were separated and discarded. Umbilical cords were carefully minced into small fragments, and the pieces were digested at 37°C for 3 h with 10 ml of 0.62 Wünsch units/mL of collagenase I. The same volume of DMEM as that of the digested fluid was added to stop the digestion, and the digested fluid was passed through a 70 μm strainer and centrifuged to collect the cells. hUCMSCs were cultured in DMDM/F12 with 10% FBS and incubated at 37°C in a humidified atmosphere with 5% CO_2_.

MSC-derived EVs were obtained from the supernatants of 10^7^ MSCs. Briefly, the supernatants were centrifugated at 2000 × *g* for 10 min, to remove cell debris. Subsequently, the supernatants were ultracentrifuged at 10,000 × *g* for 30 min, to discard microparticles, and underwent a second ultracentrifugation at 100,000 × *g* for 70 min. The pellets were suspended and stored at − 80°C.

### TEM

The morphology of isolated EVs was assessed by TEM. Briefly, EVs were blotted for 5 min onto glow-discharged 200-mesh formvar carbon-coated copper grids (Electron Microscopy Sciences, Hatfield, PA, United States). Subsequently, EVs were washed with water, followed by fixation with 2.5% glutaraldehyde in PBS. After further washing with water, the samples were stained using 2% uranyl acetate for 1 min. Negative-stained NVs were observed on an electron microscope (G2 spititi, FEI).

### Nanoparticle Tracking Analysis

The concentration and size distribution of the isolated EVs were assessed by nanoparticle tracking analysis. Briefly, NVs (10 μg/mL by BCA) were dispersed in PBS, and the particle concentration of NVs was assessed using a ZetaView Particle Metrix instrument (ZetaView PMX 110, Particle Metrix GmbH, Meerbusch, Germany). The measurements were performed in triplicate, and each individual datum was acquired from two stationary layers with five measurements from each layer. The sensitivity of the camera was configured at 70 in all measurements.

### EVs Labeling and Tissue Distribution

MSC-derived EVs were labeled with PKH26 according to the manufacturer’s instructions (Sigma). Labeled EVs (10^7^) were intravenously injected into mice. Mice were sacrificed 30 min after the injection. The distribution of EVs was evaluated in 5 μm brain sections by immunofluorescence staining with an anti-Iba1 antibody.

### Knockdown of IL-10 in MSCs

A lentiviral vector containing the IL-10 shRNA and puromycin cloning were constructed by GeneChem. MSC medium containing 8 μg ml^–1^ polybrene and the virus at an MOI of 10 were added to the cells. Half of the medium was changed every 24 h. Infected MSCs were selected by continuous incubation in MSC medium containing 10 μg ml^–1^ puromycin (Sigma), starting 1 day after transduction.

### Focal Brain Ischemia

The induction of focal cortical stroke was performed as described previously (Wendeln et al., 2018). Briefly, mice were anesthetized with 4% isoflurane in 30% O_2_ and 70% N_2_ and maintained on 2% isoflurane in 30% O_2_ and 70% N_2_ using a mask. Three-month-old mice were fixed in a stereotactic frame and a circular piece of skull was removed (5 mm in diameter, centered on Bregma). The dura mater was carefully removed with the help of a microhook (Fine Science Tools) and 5 μl of ET-1 (Bachem; 64 μM) in Hanks buffered salt solution (Invitrogen) or vehicle solution was topically applied to the cortex and incubated for 10 min. The craniotomy was then covered with a 5 mm glass coverslip, which was fixed in place with dental cement (Hybond). The skin was sutured and the mice received adrenergic receptor antagonists (flumazenil and atipamezole: 0.5 and 2.5 mg/kg of body weight, respectively) and their health was monitored. Control mice underwent the same surgical procedure with application of vehicle solution to the cortex.

### Western Blot Analysis

Samples were lysed in RIPA buffer (50 mM Tris–HCl, pH 7.5, 150 mM NaCl, 1% Triton X-100, 0.1% SDS, 0.5% deoxycholate) with phosphatase and protease inhibitors (Thermo Fisher Scientific). EVs (25 μg) and whole-cell lysates (25 μg) were subjected to SDS−PAGE and transfected to a polyvinylidene difluoride membrane. The membrane was blocked with non-fat milk for 1 h and then incubated with primary antibodies overnight at 4°C. After washing with TBST, the membranes were incubated with the secondary antibodies for 1 h and then subjected to chemiluminescence analysis.

The following primary antibodies were used: rabbit anti-CD9 (1:1000; 13403, Cell Signaling Technology, United States), rabbit anti-CD29 (1:1000; 4706, Cell Signaling Technology, United States), rabbit anti-CD63 (1:1000; 55051, Cell Signaling Technology, United States), mouse anti-CD105 (1:1000; 14606, Cell Signaling Technology, United States), rabbit anti-CD73 (1:1000; 13160, Cell Signaling Technology, United States), rabbit anti-GAPDH (1:1000; 5174, Cell Signaling Technology, United States).

The following secondary antibodies were used: goat-anti-rabbit HRP-conjugated antibody (1:1000; Cell Signaling Technology, United States) and goat-anti-mouse HRP-conjugated antibody (1:1000; Cell Signaling Technology, United States).

### Immunostaining

Immunohistochemical staining was performed on free-floating sections using either the Mouse or Rabbit Specific HRP/DAB IHC Detection Kit (ab236466, Abcam, United States) or fluorescent secondary antibodies (Abcam, United States). Unless otherwise noted, brain sections were blocked for 1 h with 5% normal serum of the species in which the secondary antibody was developed, followed by incubation with the primary antibody overnight at 4°C. Sections were then washed and incubated with secondary antibodies. Cresyl violet staining was performed according to the manufacturer’s instructions.

The following primary antibodies were used: rabbit anti-Iba1 (1:500; ab5076, Abcam, United States) and mouse anti-Aβ (1:1,000; SIG-39320, BioLegend, United States).

### Stereological Quantification

Stereological quantification of neuronal damage and microglia was performed by a blinded observer on random sets of every 200 μm systematically sections throughout the neocortex. Analysis was conducted using the Image J (NIH, United States).

### ELISA

The quantification of Aβ by ELISA (Meso Scale Discovery) in brain homogenates was performed according to the manufacturer’s instructions. Briefly, samples were pre-treated with 70% of formic acid (Sigma-Aldrich), sonicated for 35 s on ice, and centrifuged at 25,000 × *g* for 1 h at 4°C. Neutralization buffer [1 M Tris base, 0.5 M Na_2_HPO_4_, and 0.05% NaN_3_ (wt/vol)] was then added at a 1:20 ratio. Aβ was measured by an observer blinded to the treatment groups using the human (6E10) Aβ triplex assay (Meso Scale Discovery, MSD).

For cytokine measurements, brain homogenates were centrifuged at 12,000 × *g* for 15 min at 4°C. The supernatants were analyzed according to the manufacturer’s instructions. To determine blood cytokine levels, the serum was obtained by coagulation of whole blood in Vacuettes (363080, BD, United States) for 10 min at room temperature and centrifugation for 10 min at 2,000 × *g*. Serum samples were diluted 1:2 before measurements. The investigator was blinded to the treatment groups.

### Statistical Analysis

Data were analyzed using SPSS Statistics 22 and GraphPad Prism 8.0 and are presented as the mean ± standard deviation. The number of experiments performed with independent mice (n) is indicated in the figure legends. Different treatment groups were evaluated using one-way analysis of variance with Tukey’s test for multiple comparisons among individual groups. A probability of *P* < 0.05 was considered indicative of a significant difference between groups. Regardless of the method used, the results are equivalent in magnitude and statistically significant. Sample size was calculated based on a power value of 0.95 and an α value of 0.05.

## Data Availability Statement

The raw data supporting the conclusions of this article will be made available by the authors, without undue reservation.

## Ethics Statement

The animal study was reviewed and approved by Ethics Committee of Fudan University, Shanghai, China.

## Author Contributions

YF and MG drafted the manuscript. YF, SH and HZ accomplished the experiment. QD and MC designed the experiment and provided financial support. All authors contributed to the article and approved the submitted version.

## Conflict of Interest

The authors declare that the research was conducted in the absence of any commercial or financial relationships that could be construed as a potential conflict of interest.
